# An unusual new species of *Hallodapomimus* Herczek, 2000 from the Eocene Baltic amber (Hemiptera, Heteroptera, Miridae, Phylinae)

**DOI:** 10.3897/zookeys.489.8886

**Published:** 2015-03-23

**Authors:** Aleksander Herczek, Yuri A. Popov

**Affiliations:** 1 Silesian University, Department of Zoology, 40-007, Bankowa 9, Katowice, Poland; 2 Borissyak Paleontological Institue, Russian Academy of Sciences (Arthropod laboratory), Profsoyuznaya str. 123, 117997, Russia

**Keywords:** Heteroptera, Miridae, Phylinae, Hallodapini, Baltic amber

## Abstract

*Hallodapomimus antennatus* **sp. n.** (Hemiptera: Heteroptera, Miridae, Phylinae, Hallodapini) is described from a macropterous female found in Eocene Baltic amber. The new species can be recognized readily from the other species of the genus, mainly due to its unusual second antennal segment. A key for the identification of all known fossil Hallodapini is presented.

## Introduction

The present article is а continuation of a series of taxonomic papers on fossil plant bugs (Miridae) from Baltic amber (Prussian Eocene Formation). Miridae represent the largest family among true bugs (Hemiptera: Heteroptera), widespread all over the world, and with approximately 1500 genera and more than 11 000 described species, with potentially thousands more undescribed (Schuh 2002–2013; Cassis and Schuh 2012; Menard et al. 2013). Most of those included in family Miridae are frequently discovered in the Eocene Baltic amber where mirids are represented mainly by the subfamilies Cylapinae, Isometopinae, Psallopinae, and Mirinae (mainly undescribed) with fewer numbers from the remaining subfamilies (Popov and Herczek 2008). The Phylinae are quite rare among amber inclusions and all species described represent the tribe Hallodapini.

The recent Phylinae is one of the numerous subfamilies of mirids currently divided into six tribes, comprising more than 300 genera among which 50 genera belong to the tribe Hallodapini. Their representatives mainly occur temperate regions but there is also a large fauna in tropical and subtropical Asia (Schuh 1995; Schuh and Menard 2013). Moreover, many phylines have a variable myrmecomorphic habitus (McGiver and Stonedahl 1993), e.g. Hallodapini, Leucopterophorini, Auricillocorini and Pilophorini.

Herczek (2000) established the new genus *Hallodapomimus* of the tribe Hallodapini with two new species: *Hallodapomimus elektrinus* (the type species of the genus) and *Hallodapomimus succinus*, both of which were found in Baltic amber. Extinct phyline species had not been previously recorded. Very little is known about the biology of recent Hallodapini, such as the way of life or ecological preferences. Later Herczeket al. (2010) established another new monotypic genus *Leptomimus* (a junior homonym) named subsequentely new name *Leptomimoides* (Herczek and Popov 2011) with a new species *Leptomimoides jonasdamzeni*; they also described another new species, *Hallodapomimus krzeminskiorum*.

## Material and methods

Colour photographs and drawings were made with a Nikon Eclipse E 600 microscope and by the computer program NIS Elements, Ver. 4. 10. Body length was measured from the apex of head to the apex of fore wing; body width, across the maximal width; pronotum length, along midline; pronotum width, across the broadest part at its posterior angles; hemelytron length, from the base to the apex of anterior margin; hemelytron width, at maximal width of the hemelytron. All measurements are in millimeters (mm).

## Systematic paleontology

### Order Hemiptera Linnaeus, 1758 Suborder Heteroptera Latreille, 1810 Infraorder Cimicomorpha Leston, Pendergrast & Southwood, 1954 Superfamily Miroidea Hahn, 1833 Family Miridae Hahn, 1833 Subfamily Phylinae Douglas & Scott, 1865 Tribe Hallodapini van Duzee, 1916

#### Genus Hallodapomimus

Taxon classificationAnimaliaHemipteraMiridae

Herczek

Hallodapomimus : Herczek 1998: 12, nomen nudum; Herczek 2000: 144; Popov and Herczek 2008: 68; Herczek et al. 2010: 585.

##### Type species by original designation.

*Hallodapomimus elektrinus* Herczek, 2000: 145.

##### Diagnosis.

Distinguished from the other extinct hallodapine genus *Leptomimoides* by a combination of the following characters: smooth, impunctate dorsal surface of body, distinctive coloration (head, pronotum and part of cuneus dark, and clavus partly black), head almost twice as broad as long, pronotum 1.2–1.3 times wider than long; pronotal calli visible.

#### Hallodapomimus antennatus

Taxon classificationAnimaliaHemipteraMiridae

Herczek & Popov sp. n.

http://zoobank.org/B1122F2D-4DC7-4F2F-A884-D0CA9BB34B3D

Figs 1 , 2–3 , 4–5

##### Type material.

Holotype: female, Baltic amber, PIN RAS 964/1310; light yellowish middle-sized piece of amber (28 × 12 mm) of irregular shape. One dipteran syninclusion. The holotype is deposited in the collection of the Borissyak Paleontological Institute Russian Academy of Sciences (Arthropod Laboratory), Moscow.

##### Diagnosis.

Readily recognized among the other species of *Hallodapomimus* by its unusual flattened and widened second antennal segment, presence of two cavities on the vertex, a small scutellum (except *Hallodapomimus succinus*), and a large mesoscutum.

##### Description.

**Figure 1.**
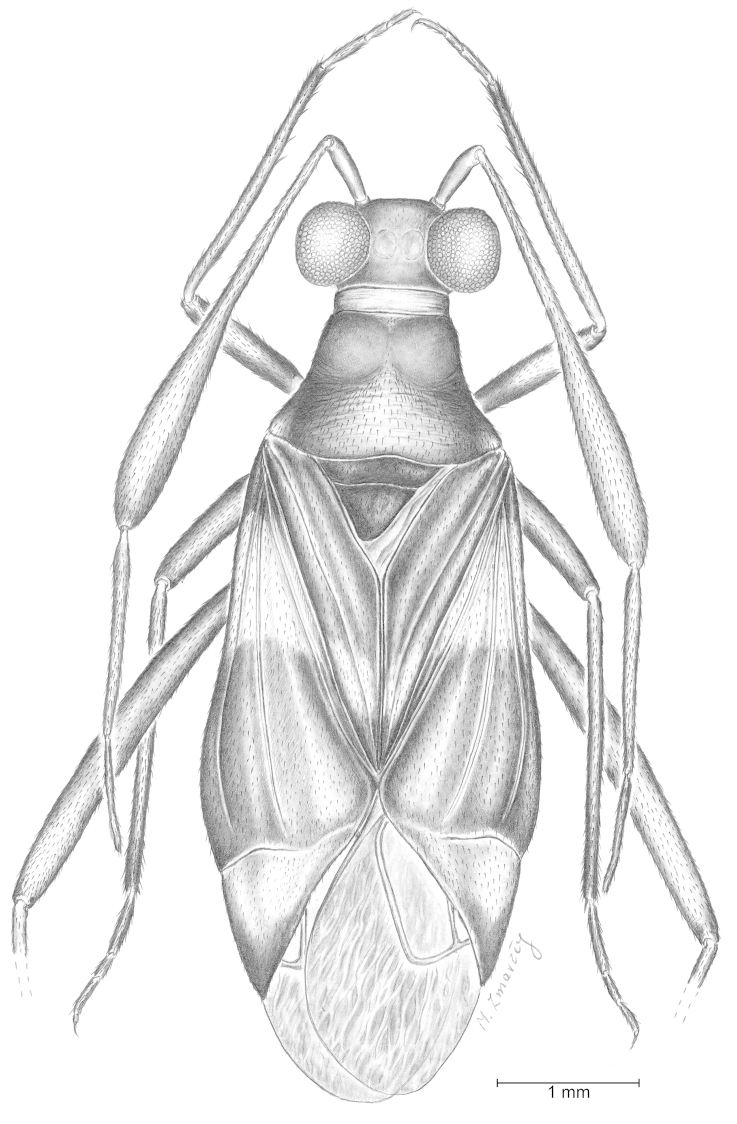
*Hallodapomimus antennatus* sp. n. ♀ holotype, in Baltic amber, nr. PIN RAS 964/1310; Borissyak Paleontological Institue, Russian Academy of Sciences. Dorsal view.

**Figures 2–3.**
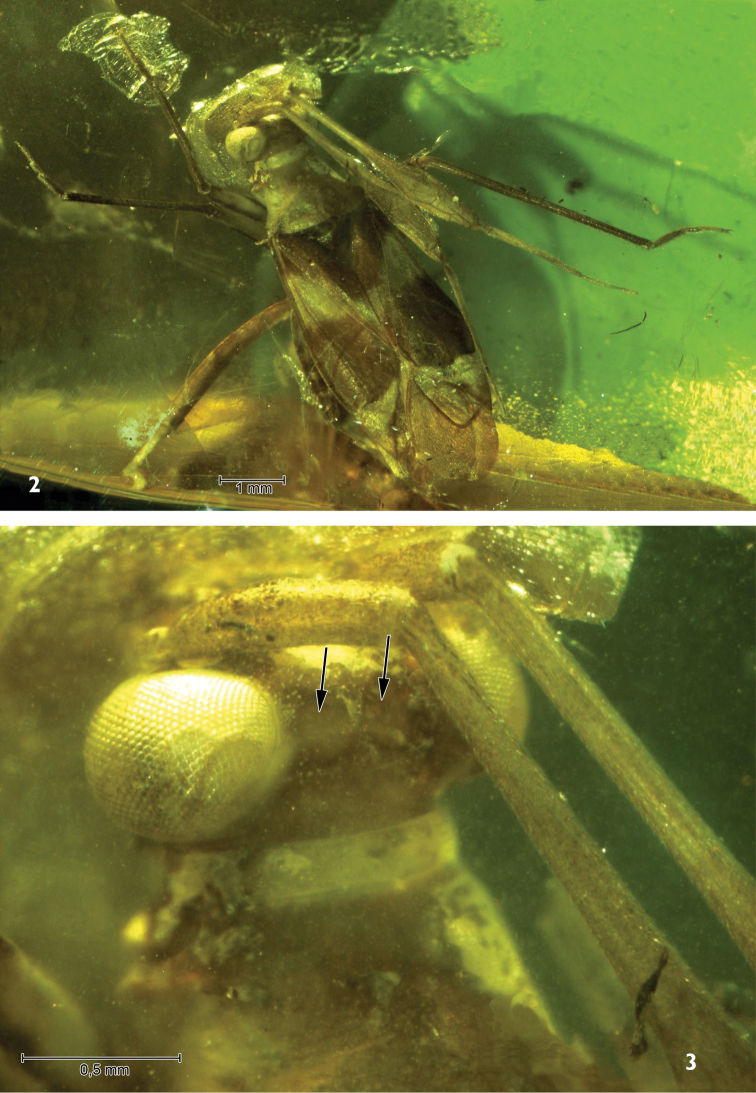
*Hallodapomimus antennatus* sp. n. **2** dorsal view **3** dorsal view of head.

**Figure 4–5.**
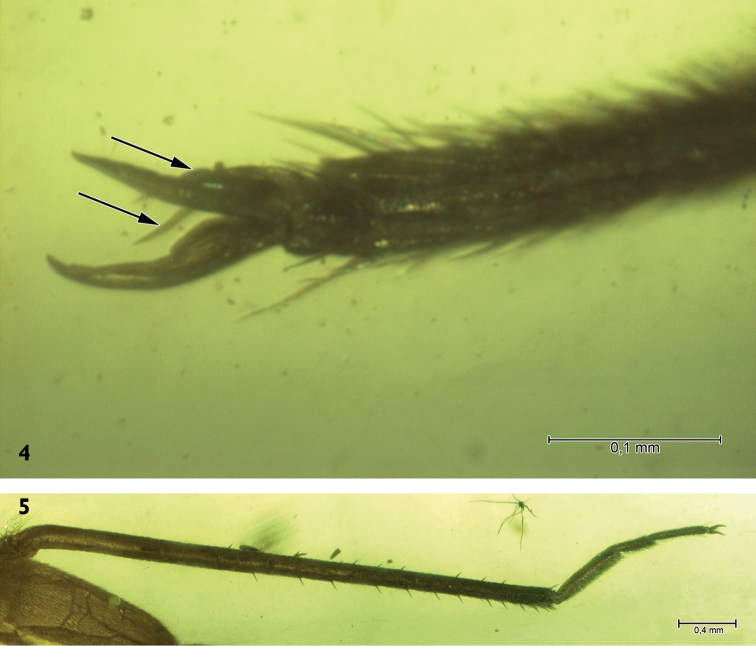
*Hallodapomimus antennatus* sp. n. **4** hind leg tibia **5** hind leg tarsus.

Female. Macropterous. Body length up to 7 mm, 2.8 times as long as wide. Dorsal surface almost smooth, impunctate. Ground colour light brown, almost yellow; mesoscutum and scutellum brown, hemelytra with one pale transverse fascia just posterior to scutellum, apical part of cuneus dark; hemelytral membrane dark, hyaline, slightly crumpled (Figs 1, 2). Head more than twice (2.3 times) as broad as long; clypeus distinct and not protruding above frons; genal conus distinct; eyes large, almost globular, distinctly protruding laterally and almost touching pronotal collar; vertex with two slightly concave, polished cavities (Fig. 3), antennae inserted just above the lower margins of eyes; fovea antennalis touching the inner margin of eye; second antennal segment laterally flattened and considerably widened to apex, 2.2 times longer than 3^rd^ segment, 3^rd^ almost twice as long as 4^th^ one; rostrum reaching hind coxae. Pronotum tapering (narrowing) to ca. 1.75 (1.76) its length, 1.37 times wider than long; collar rather broad, flat; calli distinctly developed, quite large, occupying almost half of pronotal disc. Mesoscutum broadly exposed, scutellum quite small, only twice longer than mesoscutum length and ca. one third length of claval commissure, distinctly convex. Hemelytra wholly flattened; cuneus rather short: ca. one third length of corium and one fifth times length of hemelytron; large cell of hemelytral membrane almost rectangular, smaller cell very small, almost 4 times shorter than large cell (Figs 1, 2). All legs rather slender and covered with very short, dense, adpressed setae; hind tibia with two rows of very short spines on dorsal (10–11) and ventral (5–6) surface of its distal part, these clearly shorter than diameter of tibia (Fig. 4); first tarsal segments longest, second shorter than third (Fig. 4); claws short and slightly curved, setiform parempodia easily visible (Fig. 5).

**Measurements.** Body length 7.0 mm, width 2.5; length of head 0.65, width 1.5; width of eye (from above) 0.65; width of vertex 0.5; length of antennal segments = 0.75: 3.65: 1.8: 0.95 (7.15 mm); length of rostral segments I: II: III: IV = 0.74: 1.17: 0.44: 0.6; length of pronotum 1.24, anterior width (collar) 0.85, posterior width 1.7; thickness of collar 0.18; length of hemelytron 4.79, width 1.16; proportion of hemelytron, corium and length of cuneus: 4.8–2.9–1.0; length of mesoscutum 0.2 (mid line 0.2), width 0.6; length of scutellum 0.4; claval commissure 1.3; hind leg: length of femora 3.0, tibia 4.2, tarsus 1.38 (0.59:0.35:0.44).

##### Etymology.

The species epithet (Latin “antennatus”) refers to the unusual flattened and widened the second antennal segment.

### Key to the Hallodapini from Baltic amber

**Table:** 

1	Body strongly elongate, more than 4 times as long as wide; dorsum of surface rippled. Head slightly more than 1.5 times as broad as long. Pronotum length and width subequal; pronotal calli indistinct. Head, pronotum and cuneus pale	***Leptomimoides jonasdamzeni* Herczek & Popov**
–	Body less than 4 times as long as wide; dorsum smooth, impunctate. Head almost twice as broad as long. Pronotum 1.2–1.3 times wider than long; calli weakly developed. Head, pronotum and part of cuneus dark	**2**
2	Second antennal segment flattened and considerably widened to apex, more than two times longer than 3^rd^; vertex with two slightly concave cavities; scutellum small, only twice long as mesoscutum length and less that one-third length of claval commissure	***Hallodapomimus antennatus* sp. n.**
–	Second antennal segment more slender, not expanded apically; less than twice as long as 3^rd^; vertex without cavities; scutellum large, ca. one-half length of claval commissure	**3**
3	Mesoscutum large, slightly more than one-half as long as scutellum; first tarsal segment of hind leg longest, second segment shortest	***Hallodapomimus succinus* Herczek**
–	Mesoscutum small, one-fifth as long as scutellum; first and third tarsal segments of hind legs longest and almost equal in size	**4**
4	Pronotal collar less narrow, thickness not less than 0.15 mm; cuneus less that one-fourth length of corium; all pairs of legs almost wholly bare	***Hallodapomimus elektrinus* Herczek**
–	Pronotal collar more narrow, thickness 0.1 mm; cuneus ca. one-third length of corium; all pairs of legs are covered with very short, dense, adpressed setae	***Hallodapomimus krzeminskiorum* Herczek & Popov**

## Supplementary Material

XML Treatment for Hallodapomimus

XML Treatment for Hallodapomimus antennatus
